# Promoting physical activity through text messages: the impact of attitude and goal priority messages

**DOI:** 10.1080/21642850.2021.1891073

**Published:** 2021-03-01

**Authors:** Tom St Quinton, Ben Morris, Martin J. Barwood, Mark Conner

**Affiliations:** a School of Social and Health Sciences, Leeds Trinity University, Leeds, UK; b Department of Psychology, University of Leeds, Leeds, UK

**Keywords:** Physical activity, health behavior, exercise psychology, theory of planned behavior, text messaging

## Abstract

**Introduction:**

Many young adults demonstrate insufficient rates of physical activity (PA) to yield health benefits. The study tested the effectiveness of a text messaging intervention targeting key psychological determinants and PA.

**Methods:**

Participants received either attitude messages, goal priority messages, a combination of these, or generic PA information (control). After confirming that groups were matched at baseline, a 2 (attitude: yes vs. no) by 2 (goal priority: yes vs. no) by 2 (time: immediately post-intervention, four weeks post-intervention) randomized control trial tested main and interactive effects.

**Results:**

Results showed participants that received attitude messages had significantly more positive attitudes, intentions and rates of PA. Mediational analyses showed the influence of attitude messages on PA to be fully mediated through the serial path via attitude and intention. There were no other main or interactive effects.

**Conclusion:**

The study provides support for using attitudinal messages delivered via text messaging to influence key psychological determinants and PA.

## Introduction

The benefits of PA are well established. For example, regular participation can reduce mortality rates and delay the onset of many chronic diseases such as cancer, diabetes, and heart disease (Ignarro, Balestrieri, & Napoli, 2007; Warburton, Nicol, & Bredin, 2006). Despite these benefits, a large proportion of the population do not meet recommended PA guidelines (Rhodes, Janssen, Bredin, Warburton, & Bauman, 2017). Participation in PA has been demonstrated to decrease through adolescence and into early adulthood (Dumith, Gigante, Domingues, & Kohl, 2011; Telama, 2009) which is problematic because those adopting PA during this period are more likely to continue participating in the future (Lee & Loke, 2005). The university setting provides an opportunity to influence PA rates of young adults (Allom, Mullan, Cowie, & Hamilton, 2016), especially as students’ perceptions towards behaviors are still being formed (Nelson, Story, Larson, Neumark-Sztainer, & Lytle, 2008). Additionally, university students spend a considerable amount of time in educational environments which promote sedentary behavior, and, in addition, are largely being educated for sedentary occupations (Fotheringham, Wonnacott, & Owen, 2000). It has been demonstrated that students are also insufficiently physically active to achieve health benefits (Haase, Steptoe, Sallis, & Wardle, 2004; Keating, Guan, Piñero, & Bridges, 2005).

### The theory of planned behavior

It has been argued that efforts to promote health behaviors should be developed on the basis of health psychological theory (Glanz & Bishop, 2010; Hagger & Weed, 2019). Theory provides insights into the psychological mechanisms of action which can facilitate intervention development (Hagger, 2009; Michie, Johnston, Francis, Hardeman, & Eccles, 2008). One of the most popular theories adopted in the health domain is the Theory of Planned Behavior (TPB; Ajzen, 1985) which suggests behavior is a consequence of four determinants. The proximal determinant of behavior is an intention which represents a person’s motivation to exert effort to perform the behavior. Intention is determined by three factors; attitude, subjective norm, and perceived behavioral control. Attitude concerns perceptions toward the behavior, whether it be favorable or unfavorable. Subjective norm refers to perceptions of social pressure from significant others to perform the behavior. Perceived behavioral control relates to the perceived ease or difficulty of performing the behavior.

Meta-analytic reviews have supported the theory in providing a good account of intention and behavior. More specifically, attitude, subjective norm and perceived behavioral control have been found to explain 40–45% of the variance in intention, and intention and perceived behavioral control to explain 19–36% of the variance in behavior (Armitage & Conner, 2001; Hagger, Chatzisarantis, & Biddle, 2002; McEachan, Conner, Taylor, & Lawton, 2011). The influence of attitude has received particular attention in relation to PA (Biddle & Mutrie, 2008) and a number of studies have found the construct to exert the greatest impact on intentions to be physically active (e.g. Hagger et al., 2002; Kwan, Bray, & Martin Ginis, 2009; Plotnikoff, Lubans, Costigan, & McCargar, 2013). For example, Plotnikoff et al. (2013) found attitude (*r* = .70) but not subjective norm (*r* = .00) and perceived behavioral control (*r* = .13) to significantly predict PA intentions. Reviews have also demonstrated medium-to-large changes in intention (Sheeran et al., 2016; Webb & Sheeran, 2006) result in small-to-medium changes in health behaviors (Hardeman et al., 2002; Webb & Sheeran, 2006), including PA (Rhodes & de Bruijn, 2013; Rhodes & Dickau, 2012). For example, Rhodes and Dickau (2012) found interventions targeting various determinants of PA to have an effect size of *d* = 0.45 on intention and *d* = 0.15 on behavior.

It is evident that strong intentions are not always translated into behavior, a discordance which is commonly known as the ‘intention-behavior gap’ (Sheeran, 2002). It is therefore of interest to examine how interventions can be developed to reduce this gap. Theories comprising post-intentional phases, volitional factors and facilitating strategies have been developed attended to this issue (see Rhodes & Yao, 2015). However, a limitation of previous attempts to facilitate intention translation is the focus on a single intention, especially when many health behaviors are part of several additional goals, intentions and behaviors that could be potentially pursued.

### Goal priority

Individuals may simultaneously hold intentions towards multiple goals, even in the health domain (e.g. to regularly participate in PA, abstain from smoking, reduce alcohol consumption). Health-related behaviors cluster among university populations and need to be taken into account when designing multi-health interventions and policies (Murphy et al., 2019). Such goals may be in conflict and it has been suggested that the prioritization of one goal over another may facilitate intention translation (Conner et al., 2016). Here it is assumed that prioritized goals are more likely to be activated and committed to than goals that are not prioritized. Thus, those who prioritize enacting their PA intentions over other goals might be more likely to implement their PA intentions.

Only four studies reported by Conner et al. (2016) have examined the influence of goal priority on health behaviors. This research comprised both predictive (studies 1 and 4) and experimental (studies 2 and 3) studies relating to single (studies 1–3) and multiple (study 4) health behaviors. Studies 1–3, which focused on PA, found intention had stronger predictions of behavior when goal priority was high. This was also replicated in a number of health protection (i.e. eating a low-fat diet) and health risk (i.e. binge drinking) behaviors (study 4). In the experimental studies (studies 2 and 4), participants were asked to write down how they would prioritize participation in PA and subsequent self-reported (study 2) and objective (study 3) measures of PA were taken. Both studies found the goal priority manipulation led to increases in goal priority and Study 2 demonstrated greater change in PA within the goal priority condition compared to a control. This series of studies provide preliminary evidence for the importance of goal priority within a number of health behaviors, including PA. More specifically, prioritizing a goal appears to strengthen the relationship between intention and behavior. However, it is not yet known whether goal priority can also be effective within other intervention delivery modalities.

### SMS delivery mode

Many modalities are available for delivering health interventions (Beck et al., 2016; Dombrowski, O'Carroll, & Williams, 2016). The adoption of mobile phones to target PA rates has gained recent popularity, particularly those using the short message service (SMS) (King et al., 2020; Legler, Celano, Beale, Hoeppner, & Huffman, 2020). A SMS, or text message, comprises a maximum of 160 characters and is distributed to a mobile phone. This mode of delivery provides many benefits for interventions targeting health behaviors. For example, SMS is relatively cheap, simple, and has high reach (Atun & Sittampalam, 2006; Horner, Agboola, Jethwani, Tan-McGrory, & Lopez, 2017). Moreover, text messages can be accessed at any time and are delivered immediately, even if a phone has been switched off (Gold, Lim, Hellard, Hocking, & Keogh, 2010). Given students’ prevalent use of mobile phones (Fowler & Noyes, 2015), interventions targeting students’ health behaviors are highly suited to this modality.

In terms of effectiveness, interventions using SMS have yielded small but positive effects on health-related behaviors (Armanasco, Miller, Fjeldsoe, & Marshall, 2017; Head, Noar, Iannarino, & Grant Harrington, 2013; Orr & King, 2015). For example, a recent meta-analysis undertaken by Head et al. (2013) reported interventions adopting SMS to have an effect size of *d* = 0.33 on health behaviors. This is also applicable to PA, with Buchholz, Wilbur, Ingram, and Fogg (2013) reporting all SMS interventions targeting the behavior to have an effect size greater than *d* = 0.20. Given the high reach of SMS interventions, these small effects can have significant impact on health behaviors (Armanasco et al., 2017; Webb, Joseph, Yardley, & Michie, 2010). Studies have also used text messages to specifically target attitudes towards PA (e.g. Mistry, Sweet, Rhodes, & Latimer-Cheung, 2015; Sirriyeh, Lawton, & Ward, 2010). The SMS delivery mode thus appears to have the potential to change important psychological determinants towards PA and subsequent participation rates.

### Purpose

PA is an important health behavior for young adults to undertake and research has established participation in PA to be influenced by the attitude construct. Research has also identified a gap between intention and behavior and the goal priority strategy has demonstrated success in strengthening the intention-behavior relationship. Despite this, as far as we are aware, no study has targeted participation in PA using text messages manipulating attitude and goal priority. Thus, the purpose of the study was to test the effectiveness of attitude and goal priority text messages in promoting students’ participation in PA. It was hypothesized that (1) attitude messages would have a main effect on attitude, intention, and PA, (2) the influence of attitude messages on PA would be mediated through attitude and intention, (3) goal priority messages would have a main effect on goal priority and PA, (4) the influence of goal priority messages on PA would be mediated through goal priority, and (5) goal priority messages would augment the effects of attitude messages on PA.

## Materials and method

### Design and procedure

The study adopted a 2 (attitude message: yes vs. no) x2 (goal priority message: yes vs. no) x2 (time: immediately post-intervention, four weeks post-intervention) factorial design. Contact lists were generated of departmental offices from many disciplines within 104 universities in the United Kingdom. Emails comprising study information and a recruitment poster were sent and they were asked to circulate the latter to their first-year students. Participants then accessed the survey by either clicking the hyperlink on the poster or copying the URL. Once accessed, further information on the study was provided and those willing to participate read and provided consent to the statements given. Participants then completed the baseline questionnaire (T0) and once complete, were informed when the intervention would commence for them. All interventions started on a Tuesday, but the precise date depended on the time of enrollment. A computer-generated random number sequence was used to allocate participants individually to one of four conditions at the point of enrollment; attitude only, goal priority only, attitude and goal priority, and control. Immediately after undertaking the intervention, participants were asked to respond to the first follow-up questionnaire (T1). Participants were then required to respond to the second follow-up questionnaire four weeks later (T2). All assessments were completed online and participants were sent text messages with links to the relevant questionnaires. Participants could either click the link or insert the URL to gain access. To match data across all three time-points, participants provided their mobile phone number and responded to three personal questions to generate a pseudo code. Ethical approval was gained from the University ethics board prior to study recruitment.

### Participants

**Figure 1.**
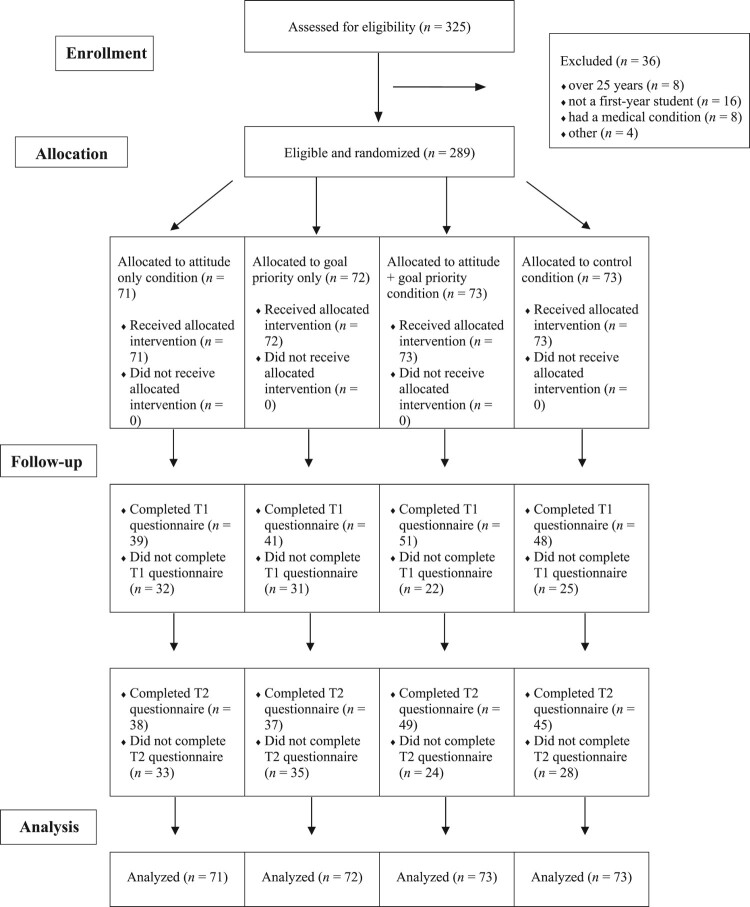
CONSORT flow diagram of study participants.

Participants were eligible to participate in the study if they were; (1) aged between 18–25 years, (2) a first-year undergraduate student, and (3) owned a mobile phone. Participants were excluded if; (1) they were currently, or had ever, taken medication for a heart condition or (2) had any medical conditions that may have affected their participation in PA. From the 325 enrolled participants, a total of 289 participants from 57 universities were eligible to participate (*n* = 106 males, 183 females; *M *= 18.7 years, *SD *= 1.17). These were randomized into one of the four intervention conditions; attitude only (*n* = 71), goal priority only (*n* = 72), attitude and goal priority (*n* = 73), and control (*n* = 73) (see Figure 1).

### The intervention

Text messages were distributed to participants using an online text messaging service which enabled messages to be scheduled and sent automatically. All intervention conditions received a total of six messages that were sent on various days (i.e. Monday, Tuesday, Thursday, Saturday) and at various times (i.e. midday, 9am, 2pm) throughout the two-week intervention period. Regardless of the condition participants were allocated, the timing of receiving a message was the same for each condition. Message content was based on previous attempts to change attitude towards PA (e.g. Conner, Rhodes, Morris, McEachan, & Lawton, 2011; Morris, Lawton, McEachan, Hurling, & Conner, 2016; Sirriyeh et al., 2010) and the use of the goal priority strategy (e.g. Conner et al., 2016). Participants reported receiving an average of 5.82 (1.35) text messages (minimum = 1 (*n* = 2); maximum = 9 (*n* = 3); mode = 6 (*n* = 82)) and 25 participants stated ‘Don’t know.’

*Attitude only*. Participants in the attitude condition received messages concerning the benefits of PA and how participation can be particularly beneficial to them as a university student. For example, participants were sent messages including ‘Physical activity can reduce the risk of a number of chronic diseases such as type 2 diabetes. Why not perform physical activity?’ and ‘Participating in physical activity throughout your period of study provides opportunities to make friends & socialize. Why not get involved in physical activity?.’

*Goal priority only*. Participants in the goal priority condition were asked to prioritize PA. Examples of goal priority messages included ‘It has been found that writing down how you will prioritize a goal can help you achieve it. Make an attempt at writing down how you will prioritize physical activity’ and ‘Realize your goal by prioritizing it. Have a go at writing down how you will prioritize physical activity.’

*Attitude and goal priority*. Those participants in the combined attitude and goal priority condition received a combination of the messages sent to the individual attitude and goal priority conditions. An example of a text message was Physical activity can reduce the risk of a number of chronic diseases such as type 2 diabetes. Why not perform physical activity? It has been found that writing down how you will prioritize a goal can help you achieve it. Make an attempt at writing down how you will prioritize physical activity.

*Control*. Participants in the control condition received text messages with generic information relating to PA (i.e. definitions of PA and recommended participation guidelines). Examples of a messages sent to the control condition include ‘Current guidelines suggest adults should perform physical activity at least 5 days per week for 30 minutes’ and ‘Physical activity is defined as any bodily movement produced by skeletal muscles that require energy expenditure.’

### Measures

To ensure the definition of PA was understood and consistent, participants were provided with the following description at each assessment time point;

Please note that we are defining physical activity as those moderate to vigorous exercise activities such as jogging, running, and cycling. We also include sports within this definition (e.g., football, rugby, tennis) and anaerobic exercises (e.g., swimming lengths), but not light exercises (e.g., walking or golf). We are referring to such activities being performed in bouts of at least 30 minutes on at least 5 days of the week over the next 2 weeks.

Full measures are provided in the supplementary file, but examples are provided below.

*Psychological constructs*. Attitude, intention, and goal priority were measured at each of the three time-points. Five items measured attitude (e.g. For me, participating in physical activity would be, Unenjoyable-Enjoyable, Cronbach’s *α* = T0: 0.81, T1: 0.80, T2: 0.85) and three items measured intention (e.g. I plan to take part in physical activity, Strongly agree-Strongly disagree, Cronbach’s *α* = T0: 0.78, T1: 0.82, T2: 0.83). Similar to measures used by Conner et al. (2016), three items assessed goal priority (e.g. Other goals and priorities will be set aside in order for me to participate in physical activity, True-False, Cronbach’s *α* = T0: 0.79, T1: 0.81, T2: 0.83). Subjective norm and perceived behavioral control were also measured at T0 using three (e.g. People who are important to me would disapprove/approve of me participating in physical activity, Would disapprove-Would approve, Cronbach’s *α* = .75) and four items (e.g. How confident are you that you can participate in physical activity, Not very confident-Very confident, Cronbach’s *α* = .81), respectively. Assessments of TPB items (i.e. attitude, intention, subjective norm, and perceived behavioral control) followed standard procedures (Ajzen, 2002). All items were measured using 7-point Likert scales which varied in direction.

*Physical activity*. PA was measured at each of the three time-points using three items (e.g. A typical week within the past 4 has consisted of physical activity being performed on at least 5 days, True-False, Cronbach’s *α* = T0: 0.90, T1: 0.93, T2: 0.93).

## Results

Data were analyzed in IBM SPSS (version 26). When necessary, items were reverse scored, meaning lower scores represented negative perceptions and higher scores represented positive perceptions of PA. Scores for each of the scale items were summed and averaged, giving one score per construct. Responses to the three PA items were standardized, before being summed and averaged into a single z-score.

### Randomization checks

To check adequate randomization between intervention conditions at baseline (T0), a MANOVA was conducted with age, attitude, subjective norm, perceived behavioral control, intention, goal priority and PA at T0 as the dependent variables and condition (attitude only, goal priority only, attitude and goal priority, and control) as the independent variable. There were no significant differences between conditions, *F*(21, 801) = 1.18; Wilks’ Λ = .91, *p* = .25; $\eta_{p}^{2}$ = .02. Chi-square tests also revealed no significant differences in gender distribution between conditions, *χ*^2^(3, *N* = 289) = 1.68, *p* = .64. This indication the randomization was successful.

### Attrition and MCAR analyses

From the 289 participants completing T0 assessments, 179 participants responded at T1 (61.94%), 169 at T2 (58.48%), and 135 participants completed all three assessments (46.71%). To check whether there were differences in demographics, psychological constructs, and PA at T0 between those completing all three assessments and those not, a MANOVA was conducted with T0 age, attitude, subjective norm, perceived behavioral control, intention, goal priority, and PA as the dependent variables and status of participation (completers and non-completers) as the independent variables. There were no significant differences between study participants who completed or did not complete all time points, *F*(7, 281) = 1.8; Wilks’ Λ = .95, *p* = .07; $\eta_{p}^{2}$ = .04. A series of chi square tests also revealed no significant differences in attrition between gender (χ^2^(1, *N* = 289) = .72, *p* = .59), condition (χ^2^(3, *N* = 289) = 5.21, *p* = .15), those receiving attitude messages (yes vs. no) (χ^2^(1, *N* = 289) = .59, *p* = .44), and those receiving goal priority messages (yes vs. no) (χ^2^(1, *N* = 289) = .28, *p* = .59). Additionally, patterns of missing data were analyzed and were found to be missing at random (*p* = .17 for Little’s MCAR test). Consequently, multiple imputation was conducted on all missing values using SPSS. Five new datasets were created using regression models including relevant baseline and post-intervention variables. Analyses were computed separately on each of the five imputed datasets. Similarities were apparent on each of the five analyses and generated values were within expected ranges. Rubin’s rules were then used to combine *F, p* and η2 values from each of the datasets. These again represented similarities with each of the individual datasets and so results are presented from the first imputation.

### Main analyses

**Figure 2.**
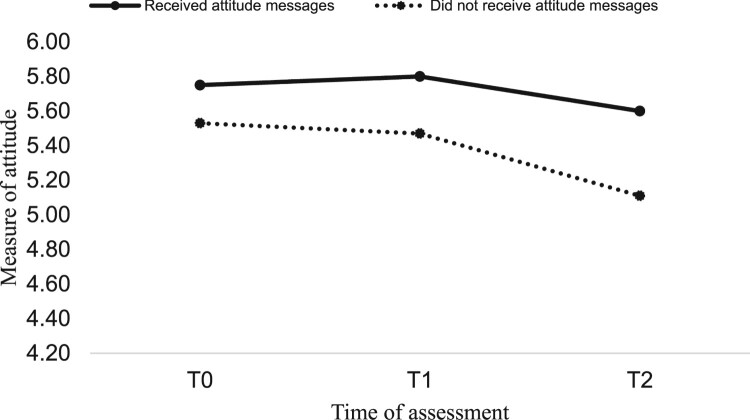
Main effect of attitude messages on attitude.

**Figure 3.**
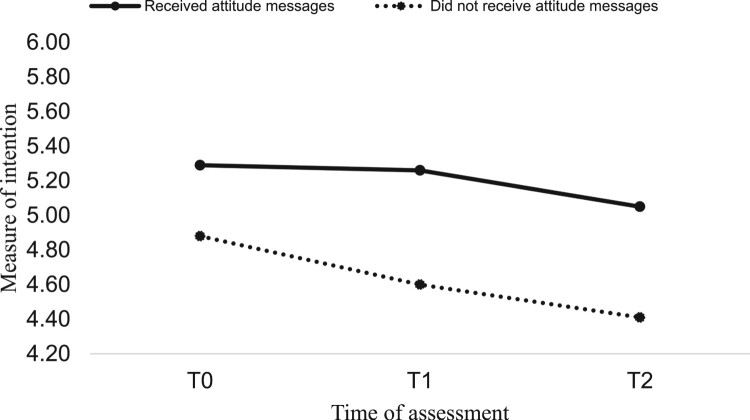
Main effect of attitude messages on intention.

**Figure 4.**
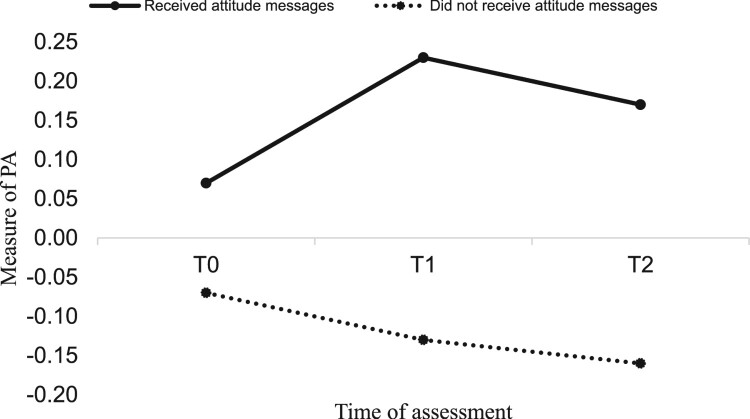
Main effect of attitude messages on PA. Note: There were no significant differences between conditions at baseline.

*Impact of attitude and goal priority messages*. To examine the impact of the messages on the psychological variables and PA, a 2 (attitude: yes vs. no) by 2 (goal priority: yes vs. no) by 2 (time: immediately post-intervention, four weeks post-intervention) mixed MANCOVA was conducted with attitude, goal priority, intention, and PA assessed immediately post-intervention (T1) and four weeks post-intervention (T2) as the (repeated-measures) dependent variables and T0 attitude, goal priority, intention, PA, age, and gender as covariates. Results showed a significant main effect for attitude messages (*F* (4, 276) = 5.76, *p* = .001, *η*^2^ = .07). Specifically, attitude messages had a significant main effect on attitude (*F* (1, 279) = 4.12, *p* = .04, *η*^2^ = .01), intention (*F* (1, 279) = 11.54, *p* = .001, *η*^2^ = .04), and PA (*F* (1, 279) = 17.06, *p* = .001, *η*^2^ = .05) (hypothesis 1). Marginal means showed participants receiving attitude messages had more positive attitudes (received = 5.64, did not receive = 5.35) and intentions (received = 5.04, did not receive = 4.62), plus greater PA (received = 0.14, did not receive = −0.08) than those that did not receive attitude messages. Goal priority messages had no main effect on the psychological constructs and PA (*F* (4, 276) = 1.85, *p* = .11, *η*^2^ = .02) (hypothesis 3) and there were no interactions between messages (*F* (4, 276) = 0.53, *p* = .70, *η*^2^ = .00) (hypothesis 4). Pooled imputed means of study variables by condition can be seen in Table 1 and significant main effects in Figures 2–4.

**Table 1.** Pooled descriptive means of attitude, goal priority, intention and PA assessed by message condition (*N* = 289).

	Attitude only (*n* = 71)	Goal priority only (*n* = 72)	Attitude & goal priority (*n* = 73)	Control (*n* = 73)	Total (*N* = 289)
Attitude					
T0	5.82 (0.97)	5.41 (1.14)	5.68 (1.19)	5.65 (1.02)	5.64 (1.09)
T1	5.90 (0.88)	5.43 (1.02)	5.71 (1.05)	5.51 (1.07)	5.63 (1.02)
T2	5.64 (1.04)	5.13 (1.20)	5.56 (1.17)	5.09 (1.23)	5.36 (1.19)
Goal priority					
T0	3.91 (1.28)	3.62 (1.34)	3.80 (1.31)	3.72 (1.30)	3.76 (1.31)
T1	4.09 (1.27)	3.88 (1.25)	4.15 (1.32)	3.78 (1.47)	3.97 (1.34)
T2	4.23 (1.22)	3.98 (1.33)	4.11 (1.30)	3.73 (1.39)	4.01 (1.32)
Intention					
T0	5.36 (1.22)	4.73 (1.76)	5.21 (1.52)	5.02 (1.46)	5.08 (1.52)
T1	5.26 (1.32)	4.72 (1.48)	5.27 (1.28)	4.48 (1.63)	4.93 (1.47)
T2	5.11 (1.32)	4.49 (1.51)	5.00 (1.21)	4.32 (1.54)	4.73 (1.44)
PA					
T0	0.12 (0.89)	−0.10 (0.94)	0.03 (0.90)	−0.05 (0.90)	0.00 (0.91)
T1	0.21 (0.85)	−0.07 (0.85)	0.24 (0.87)	−0.19 (0.90)	0.04 (0.89)
T2	0.11 (0.88)	−0.15 (0.83)	0.23 (0.81)	−0.17 (0.88)	0.00 (0.87)

*Mediation analyses*. Mediation was undertaken to establish whether changes in attitude and intention at T1 mediated the effects of attitude messages on PA at T2. The serial multiple mediator model (model 6) within the SPSS macro PROCESS was used to examine the causal chain linking the mediators (Hayes, 2017). More specifically, the analyses examined the influence of (a) attitude messages on T2 PA through T1 attitude (indirect effect 1), (b) attitude messages on T2 PA through T1 intention (indirect effect 2) and (c) attitude messages on T2 PA through T1 attitude and T1 intention, with T1 attitude influencing T1 intention (indirect effect 3). Attitude messages were entered as the independent variable, T2 PA the dependent variable, and T1 attitude and T1 intention the mediators. Thus, in accordance with the TPB, the model tested the model – attitude messages > T1 attitude > T1 intention > T2 PA. As recommended by Hayes (2017), a bootstrapping method was used to examine indirect effects with data resampled 5,000 times and 95% bias-corrected confidence intervals provided. An indirect effect and the difference between two indirect effects is established when the confidence interval does not contain zero. This procedure was undertaken separately on all of the five imputed datasets. Results were similar across all five imputations and so the findings from one imputation are presented here.

**Figure 5.**
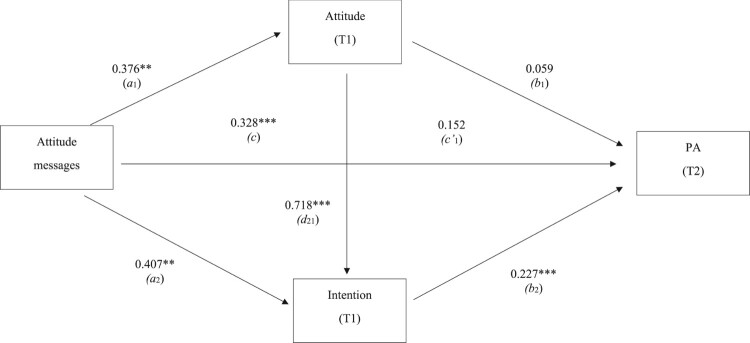
A statistical diagram of the serial multiple mediator model for the impact of attitude messages on PA through attitude and intention. Note: **p < 0.01, ***p < 0.001.

Results showed attitude messages significantly predicted T1 attitude (*a*_1_), T1 intention (*a*_2_) and T2 PA (*c*). T1 attitude significantly predicted T1 intention (*d*_21_) and T1 intention significantly predicted T2 PA (*b*_2_). Attitude messages did not significantly predict T2 PA when controlling for T1 attitude and T1 intention ($c_{1}^{\prime }$) and T2 PA was not significantly predicted by T1 attitude (*b*_1_). A statistical diagram of the serial multiple mediator model is shown in Figure 5.

The mediation analyses showed the indirect effect of attitude to be nonsignificant as the 95% bias-corrected bootstrap CI straddled zero (*a*_1_*b*_1 _= 0.0223, CI = −0.0125–0.0705). The indirect effects of both intention (*a*_2_*b*_2 _= 0.0925, CI = 0.0237–0.1727) and attitude and intention (*a*_1_*d*_21_*b*_2 _= 0.0614, CI = 0.0220–0.1057) were significantly positive as the 95% bias-corrected bootstrap CI did not straddle zero. Thus, the impact of attitude messages was mediated by the intention (indirect effect 2) and the attitude and intention (indirect effect 3) paths (hypothesis 2). No indirect effect was stronger than any other.

## Discussion

The purpose of the study was to examine the effectiveness of a SMS intervention including attitude messages and goal priority messages targeting students’ participation in PA.

### Attitude messages

In line with hypothesis 1, attitude messages had a significant influence on attitude, intention, and PA and in accordance with hypothesis 2, the effects of the messages on PA were mediated by attitude and intention. Although these effects were small, changes in the psychological constructs are not surprising given participants were students undergoing significant lifestyle transitions and adapting to university life. Indeed, this transitional period represents an ideal opportunity for health interventions to intervene as students’ perceptions towards behaviors are yet to be formed and are more amendable to change (Allom et al., 2016; Nelson et al., 2008). Changes in attitude were also unsurprising given the text messages targeting this construct were tailored towards PA. Providing the benefits of PA have been shown to influence attitudes towards the behavior within text messages (Sirriyeh et al., 2010) and other modalities (Conner et al., 2011; Morris et al., 2016). The study therefore provides evidence that the SMS delivery mode can also be adopted to manipulate attitudes towards PA and other key TPB determinants within a university sample. These findings are highly useful for interventions promoting students’ rates of PA given the ease at which SMS can be distributed and the large proportion of students in possession of a mobile phone (Fowler & Noyes, 2015; Horner et al., 2017). The mediation analyses suggested the influence of attitude messages was fully mediated by the attitude and intention path. This is in accordance with the TPB which states changes in attitude leads to changes in intention which results in behavior change (Ajzen, 1985). Thus, the study also supports the TPB’s causal mechanisms through which interventions exert influence on behavior and suggests attitude to be particularly important in influencing PA.

### Goal priority messages

Contrary to hypotheses 3–5, the study did not change goal priority and therefore could not test the effects on PA. There are a number of potential explanations for the lack of success for goal priority manipulations. It has been suggested that interventions adopting the text messaging delivery mode are sometimes less effective than other modalities (Fjeldsoe, Neuhaus, Winkler, & Eakin, 2011). Thus, it could be that goal priority is less suited to interventions delivered through SMS. Alternatively, null findings could be attributed to the various characteristics involved within text messaging interventions. There is no one-size-fits-all approach to delivering SMS interventions and message effectiveness can vary depending on the frequency, duration, and timing of messages as well as the levels of interactivity (Muntaner, Vidal-Conti, & Palou, 2016). For example, although participants received three messages per week in the current study, text messages delivered more frequently have demonstrated greater effectiveness (e.g. Franklin, Waller, Pagliari, & Greene, 2006; Orr & King, 2015). It could be that messages were too infrequent to yield any goal priority effects. Moreover, the intervention period lasted two weeks and messages were not tailored to participants. Armanasco et al. (2017) found interventions conducted over a longer period of time (i.e. 6–12 months) to be more effective and Head et al. (2013) showed the effectiveness of tailored text messages in changing health behaviors. Future research is needed to ascertain the optimal characteristics of SMS interventions targeting goal priority. Finally, although participants were instructed to prioritize PA, it seems unlikely, given the sample of study, that this was undertaken by all participants. The null findings may have therefore been a consequence of participants not engaging in the goal priority strategy. It is also worth noting that a post-hoc power calculation suggested the study was sufficiently powered to detect small-to-medium effects.

### Strengths and limitations

There are a number of strengths attached to the study. First, the intervention adopted health psychological theory and sought to address both motivation and intention translation. Due to the importance of adopting such theory and recent attention afforded to bridge the intention-behavior gap, the study was thus timely and important. Second, the study utilized a simple, yet novel implemental strategy that has received little attention to date. Third, the intervention was undertaken using a relevant and cost-effective delivery mode that was able to reach a considerable number of participants. Fourth, the intervention targeted an important health behavior within a population where declines are often seen (Bray & Kwan, 2006). Finally, the study recruited from many universities within the United Kingdom and may therefore be generalizability to other institutions.

Despite these strengths, the study was not without limitations. First, although a significant number of universities were targeted for recruitment, the response rate for participation was low. Second, the study had low rates of retention throughout each assessment timepoint. Third, despite assessing SMS delivery, we could not determine whether messages had been read. Fourth, a relatively short follow-up period was used, and changes may not have been maintained over time. Indeed, it has been recently acknowledged that initial behavior change is not synonymous with behavior maintenance (Kwasnicka, Dombrowski, White, & Sniehotta, 2016). Fifth, the length of the text messages, especially in the combined condition, sometimes exceeded the 160 characters allowed in a single message. Such messages were therefore sent over multiple messages which may have influenced the effectiveness. Finally, self-report was used to measure PA and due to recall errors and social desirability bias (Althubaiti, 2016), this method of assessment may not have provided valid accounts of PA and could have inflated relations with the psychological constructs (Plotnikoff, Lubans, Penfold, & Courneya, 2014).

### Future directions

There are a number of recommendations from the study. First, research promoting PA could adopt similar text messages to the attitude manipulations used here. Second, research is needed into identifying the most effective ways to influence goal priority (Conner et al., 2016). In relation to the SMS delivery mode, the timing and number of messages could be relevant, as could the length, tailoring, direction, and frequency. Other uses of mobile phones (i.e. mobile applications, email, voice notes) and alternative delivery modes (i.e. websites, printed materials) should also be tested. Third, as the study only targeted the attitude construct to tap into motivational processes, research could also undertake manipulations of subjective norm and perceived behavioral control (either uniquely or in combination) along with goal priority manipulations. Finally, SMS studies with longer follow-up periods and objective assessments of PA should be undertaken, and research should establish the best ways to recruit participants to studies adopting the SMS delivery mode.

## Conclusion

The study examined the effectiveness of attitude and goal priority SMS in changing key psychological mechanisms and PA. Attitude messages successfully influenced attitude, intention and behavior, and changes in behavior were mediated by changes in attitude and intention, with attitude influencing intention. The study therefore supports the TPB’s causal mechanisms through which interventions exert influence on behavior and suggests attitude to be a prominent driver of intention and subsequent PA behavior change. The study did not manage to manipulate goal priority. Future research should make use of the SMS delivery method in addressing motivational and implemental issues towards PA whilst also considering different delivery characteristics influencing its effectiveness.

## Supplementary Material

Supplemental Material

## Data Availability

The data that support the findings of this study are available from the corresponding author, TSQ, upon reasonable request.
