# Supplementary material for: Promoting physical activity through text messages: the impact of attitude and goal priority messages
Source: Health Psychol Behav Med. 2021 Mar 1;9(1):165–81. doi: 10.1080/21642850.2021.1891073 (PMC8158195; doi:10.1080/21642850.2021.1891073)
Supplement: Supplemental Material [file RHPB_A_1891073_SM7452.docx]

**Supplementary file**

**Study measures**

| **T0** | | | | | | | | |
| --- | --- | --- | --- | --- | --- | --- | --- | --- |
| **Attitude:** | | | | | | | | |
| For me, participating in physical activity would be…. | | | | | | | | |
|  | 1 | 2 | 3 | 4 | 5 | 6 | 7 |  |
| Bad | |  |  |  |  | Good | |  |
|  | 1 | 2 | 3 | 4 | 5 | 6 | 7 |  |
| Pleasant | |  |  |  |  | Unpleasant | |  |
|  | 1 | 2 | 3 | 4 | 5 | 6 | 7 |  |
| Unhealthy | |  |  |  |  | Healthy | |  |
|  | 1 | 2 | 3 | 4 | 5 | 6 | 7 |  |
| Desirable | |  |  |  |  | Undesirable | |  |
|  | 1 | 2 | 3 | 4 | 5 | 6 | 7 |  |
| Unenjoyable | |  |  |  |  | Enjoyable | |  |
| **Goal priority:** | | | | | | | |  |
| I would be prepared to give up many other goals and priorities to participate in physical activity | | | | | | | |  |
|  | 1 | 2 | 3 | 4 | 5 | 6 | 7 |  |
| Strongly disagree | |  |  |  |  | Strongly agree | |  |
| To enable me to participate in physical activity, I would be willing to sacrifice other goals and priorities | | | | | | | |  |
|  | 1 | 2 | 3 | 4 | 5 | 6 | 7 |  |
| Strongly disagree | |  |  |  |  | Strongly agree | |  |
| Other goals and priorities will be set aside in order for me to participate in physical activity | | | | | | | |  |
|  | 1 | 2 | 3 | 4 | 5 | 6 | 7 |  |
| True | |  |  |  |  | False | |  |
| **Intention:** | | | | | | | |  |
| I intend to participate in physical activity | | | | | | | |  |
|  | 1 | 2 | 3 | 4 | 5 | 6 | 7 |  |
| Strongly disagree | |  |  |  |  | Strongly agree | |  |
| How likely is it that you would participate in physical activity | | | | | | | |  |
|  | 1 | 2 | 3 | 4 | 5 | 6 | 7 |  |
| Very unlikely | |  |  |  |  | Very likely | |  |
| I plan to take part in physical activity | | | | | | | |  |
|  | 1 | 2 | 3 | 4 | 5 | 6 | 7 |  |
| Agree | |  |  |  |  | Disagree | |  |
| **PBC:** | | | | | | | |  |
| For me, participating in physical activity would be | | | | | | | |  |
|  | 1 | 2 | 3 | 4 | 5 | 6 | 7 |  |
| Very difficult | |  |  |  |  | Very easy | |  |
| How confident are you that you can participate in physical activity | | | | | | | |  |
|  | 1 | 2 | 3 | 4 | 5 | 6 | 7 |  |
| Not very confident | |  |  |  |  | Very confident | |  |
| I believe I have the ability to participate in physical activity | | | | | | | |  |
|  | 1 | 2 | 3 | 4 | 5 | 6 | 7 |  |
| Definitely do not | |  |  |  |  | Definitely do | |  |
| Factors outside my control will influence whether or not I participate in physical activity | | | | | | | |  |
|  | 1 | 2 | 3 | 4 | 5 | 6 | 7 |  |
| Strongly agree | |  |  |  |  | Strongly disagree | |  |
| **SN:** |  |  |  |  |  |  |  |  |
| People who are important to me would disapprove/approve of me participating in physical activity | | | | | | | |  |
|  | 1 | 2 | 3 | 4 | 5 | 6 | 7 |  |
| Would disapprove | |  |  |  |  | Would approve | |  |
| People who are like me will participate in physical activity | | | | | | | |  |
|  | 1 | 2 | 3 | 4 | 5 | 6 | 7 |  |
| Completely false | |  |  |  |  | Completely true | |  |
| People close to me think I definitely should not/should participate in physical activity | | | | | | | |  |
|  | 1 | 2 | 3 | 4 | 5 | 6 | 7 |  |
| Definitely should not | |  |  |  |  | Definitely should | |  |
| **Past behaviour:** | | | | | | | |  |
| A typical week within the past 4 has consisted of physical activity being performed on at least 5 days | | | | | | | |  |
|  | 1 | 2 | 3 | 4 | 5 | 6 | 7 |  |
| True | |  |  |  |  | False | |  |
| Over the past 4 weeks, on average how many days have you participated in physical activity during: | | | | | | | |  |
|  | Week 1 _______ |  |  |  |  |  |  |  |
|  | Week 2 _______ |  |  |  |  |  |  |  |
|  | Week 3 _______ |  |  |  |  |  |  |  |
|  | Week 4 _______ |  |  |  |  |  |  |  |
| During the past 4 weeks I have participated in physical activity ___ days per week on average | | | | | | | |  |
| **T1** | | | | | | | |  |
| **Attitude:** | | | | | | | |  |
| For me, participating in physical activity would be…. | | | | | | | |  |
|  | 1 | 2 | 3 | 4 | 5 | 6 | 7 |  |
| Bad | |  |  |  |  | Good | |  |
|  | 1 | 2 | 3 | 4 | 5 | 6 | 7 |  |
| Pleasant | |  |  |  |  | Unpleasant | |  |
|  | 1 | 2 | 3 | 4 | 5 | 6 | 7 |  |
| Unhealthy | |  |  |  |  | Healthy | |  |
|  | 1 | 2 | 3 | 4 | 5 | 6 | 7 |  |
| Desirable | |  |  |  |  | Undesirable | |  |
|  | 1 | 2 | 3 | 4 | 5 | 6 | 7 |  |
| Unenjoyable | |  |  |  |  | Enjoyable | |  |
| **Goal priority:** | | | | | | | |  |
| I would be prepared to give up many other goals and priorities to participate in physical activity | | | | | | | |  |
|  | 1 | 2 | 3 | 4 | 5 | 6 | 7 |  |
| Strongly disagree | |  |  |  |  | Strongly agree | |  |
| To enable me to participate in physical activity, I would be willing to sacrifice other goals and priorities | | | | | | | |  |
|  | 1 | 2 | 3 | 4 | 5 | 6 | 7 |  |
| Strongly disagree | |  |  |  |  | Strongly agree | |  |
| Other goals and priorities will be set aside in order for me to participate in physical activity | | | | | | | |  |
|  | 1 | 2 | 3 | 4 | 5 | 6 | 7 |  |
| True | |  |  |  |  | False | |  |
| **Intention:** | | | | | | | |  |
| I intend to participate in physical activity | | | | | | | |  |
|  | 1 | 2 | 3 | 4 | 5 | 6 | 7 |  |
| Strongly disagree | |  |  |  |  | Strongly agree | |  |
| How likely is it that you would participate in physical activity | | | | | | | |  |
|  | 1 | 2 | 3 | 4 | 5 | 6 | 7 |  |
| Very unlikely | |  |  |  |  | Very likely | |  |
| I plan to take part in physical activity | | | | | | | |  |
|  | 1 | 2 | 3 | 4 | 5 | 6 | 7 |  |
| Agree | |  |  |  |  | Disagree | |  |
| **Past behaviour:** | | | | | | | |  |
| A typical week within the past 2 has consisted of physical activity being performed on at least 5 days | | | | | | | |  |
|  | 1 | 2 | 3 | 4 | 5 | 6 | 7 |  |
| True | |  |  |  |  | False | |  |
| Over the past 2 weeks, on average how many days have you participated in physical activity during: | | | | | | | |  |
|  | Week 1 ___ |  |  |  |  |  |  |  |
|  | Week 2 ___ |  |  |  |  |  |  |  |
| During the past 2 weeks I have participated in physical activity ___ days per week on average | | | | | | | |  |
| **T2** | | | | | | | |  |
| **Attitude:** | | | | | | | |  |
| For me, participating in physical activity would be…. | | | | | | | |  |
|  | 1 | 2 | 3 | 4 | 5 | 6 | 7 |  |
| Bad | |  |  |  |  | Good | |  |
|  | 1 | 2 | 3 | 4 | 5 | 6 | 7 |  |
| Pleasant | |  |  |  |  | Unpleasant | |  |
|  | 1 | 2 | 3 | 4 | 5 | 6 | 7 |  |
| Unhealthy | |  |  |  |  | Healthy | |  |
|  | 1 | 2 | 3 | 4 | 5 | 6 | 7 |  |
| Desirable | |  |  |  |  | Undesirable | |  |
|  | 1 | 2 | 3 | 4 | 5 | 6 | 7 |  |
| Unenjoyable | |  |  |  |  | Enjoyable | |  |
| **Goal priority:** | | | | | | | |  |
| I would be prepared to give up many other goals and priorities to participate in physical activity | | | | | | | |  |
|  | 1 | 2 | 3 | 4 | 5 | 6 | 7 |  |
| Strongly disagree | |  |  |  |  | Strongly agree | |  |
| To enable me to participate in physical activity, I would be willing to sacrifice other goals and priorities | | | | | | | |  |
|  | 1 | 2 | 3 | 4 | 5 | 6 | 7 |  |
| Strongly disagree | |  |  |  |  | Strongly agree | |  |
| Other goals and priorities will be set aside in order for me to participate in physical activity | | | | | | | |  |
|  | 1 | 2 | 3 | 4 | 5 | 6 | 7 |  |
| True | |  |  |  |  | False | |  |
| **Intention:** | | | | | | | |  |
| I intend to participate in physical activity | | | | | | | |  |
|  | 1 | 2 | 3 | 4 | 5 | 6 | 7 |  |
| Strongly disagree | |  |  |  |  | Strongly agree | |  |
| How likely is it that you would participate in physical activity | | | | | | | |  |
|  | 1 | 2 | 3 | 4 | 5 | 6 | 7 |  |
| Very unlikely | |  |  |  |  | Very likely | |  |
| I plan to take part in physical activity | | | | | | | |  |
|  | 1 | 2 | 3 | 4 | 5 | 6 | 7 |  |
| Agree | |  |  |  |  | Disagree | |  |
| **Past behaviour:** | | | | | | | |  |
| A typical week within the past 4 has consisted of physical activity being performed on at least 5 days | | | | | | | |  |
|  | 1 | 2 | 3 | 4 | 5 | 6 | 7 |  |
| True | |  |  |  |  | False | |  |
| Over the past 4 weeks, on average how many days have you participated in physical activity during: | | | | | | | |  |
|  | Week 1 ___ |  |  |  |  |  |  |  |
|  | Week 2 ___ |  |  |  |  |  |  |  |
|  | Week 3 ___ |  |  |  |  |  |  |  |
|  | Week 4 ___ |  |  |  |  |  |  |  |
| During the past 4 weeks I have participated in physical activity ___ days per week on average | | | | | | | |  |
| **Delivery:** | | | | | | | |  |
| How many text messages did you receive throughout the intervention? Please do not include questionnaire links and prompts | | | | | | | |  |
|  | Don’t know, 0, 1, 2, 3, 4, 5, 6, 7, 8, 9, 10, other (please specify) | | | | |  |  |  |
